# *Cryptococcus neoformans* Induces MCP-1 Release and Delays the Death of Human Mast Cells

**DOI:** 10.3389/fcimb.2019.00289

**Published:** 2019-08-13

**Authors:** José Pedro Lopes, Marios Stylianou, Emelie Backman, Sandra Holmberg, Maria Ekoff, Gunnar Nilsson, Constantin F. Urban

**Affiliations:** ^1^Department of Clinical Microbiology, Umeå University, Umeå, Sweden; ^2^Umeå Centre for Microbial Research, Umeå University, Umeå, Sweden; ^3^Laboratory for Molecular Infection Medicine Sweden, Umeå University, Umeå, Sweden; ^4^Immunology and Allergy Division, Department of Medicine Solna, Karolinska Institutet, Karolinska University Hospital, Stockholm, Sweden

**Keywords:** innate immunity, *Cryptococcus neoformans*, mast cells, monocytes, monocyte chemoattractant protein 1/CCL-2, fungi

## Abstract

Cryptococcosis, caused by the basidiomycete *Cryptococcus neoformans*, is a life-threatening disease affecting approximately one million people per year worldwide. Infection can occur when *C. neoformans* cells are inhaled by immunocompromised people. In order to establish infection, the yeast must bypass recognition and clearance by immune cells guarding the tissue. Using *in vitro* infections, we characterized the role of mast cells (MCs) in cryptococcosis. We found that MCs recognize *C. neoformans* and release inflammatory mediators such as tryptase and cytokines. From the latter group MCs released mainly CCL-2/MCP-1, a strong chemoattractant for monocytic cells. We demonstrated that supernatants of infected MCs recruit monocytes but not neutrophils. During infection with *C. neoformans*, MCs have a limited ability to kill the yeast depending on the serotype. *C. neoformans*, in turn, modulates the lifespan of MCs both, by presence of its polysaccharide capsule and by secreting soluble modulators. Taken together, MCs might have important contributions to fungal clearance during early stages of cryptocococis where these cells regulate recruitment of monocytes to mucosal tissues.

## Introduction

*Cryptococcus neoformans* is an encapsulated yeast, ubiquitous in the environment (Goldman et al., 2001). Infections with *C. neoformans* are commonly diagnosed in immunocompromised patients (Singh et al., 2008; Pappas, 2013; Ventura Aguiar et al., 2014) and with the onset of the HIV/AIDS epidemic, cryptococcosis became a leading cause of death in these patients mainly due to fatal meningoencephalitis (Park et al., 2009). The infectious process begins when the host inhales aerosolized cryptococcal cells. Some particles find their way into the alveolus where the resident cells of the innate immune system attempt to clear the infection. A large body of research shows an important role for alveolar macrophages in anti-cryptococcal defense by detecting and engulfing fungal spores (Giles et al., 2009; Mansour et al., 2014). Non-professional phagocytes, such as eosinophils and mast cells (MCs) have received less attention, although one report describes effector contribution of eosinophils and interaction of MCs and *C. neoformans* (Feldmesser et al., 1997). MCs contribute to immune defense against *Aspergillus fumigatus* and *Candida albicans* (Urb et al., 2009; Trevisan et al., 2014) and orchestrate a specific and temporal response by inducing inflammatory processes in the tissue (Lopes et al., 2015; Moretti et al., 2017). To induce inflammation, MCs take advantage of their ability to recognize fungi using pattern recognition receptors (PRR) (Yang and Marshall, 2009; Agier et al., 2018) which in turn triggers the release of granular vesicles containing a broad spectrum of inflammatory mediators, cytokines, and growth factors (Mukai et al., 2018).

Our hypothesis is that the outcome of cryptococcal infection is dependent on the initial interaction with resident immune cells of the mucosal tissue, such as MCs. We, therefore, decided to explore, the interaction of MCs and *C. neoformans* using *in vitro* infections of both, a cell line and primary mast cells. We further used capsular and acapsular *C. neoformans* strains to determine the importance of capsule to the infection outcome.

## Materials and Methods

### Microbial Culture Conditions

The strains used in this study were DSM 11959 (*C. neoformans var. grubii*), B-3501 (*C*. *neoformans* var. *neoformans*), and acapsular mutant cap59 (Zaragoza et al., 2008). In all cases, microbial cells were incubated overnight at 30°C in synthetic complete dropout medium + 2% glucose. A fresh subculture was inoculated before each experiment at 30°C for 3 h.

### Immune Cell Culture Conditions

Human mast cell line 1 (HMC-1) and cord blood-derived mast cells (CBMC) were maintained and isolated as previously described (Lopes et al., 2015). Prior to any assays, MCs were primed with 25 nM PMA (12-myristate-13-acetate, Sigma-Aldrich) for 15 min at 37 °C, subsequently centrifuged for 10 min at 300 × g and re-suspended in RPMI without fetal calf serum or antibiotics. For spleen tyrosine kinase (Syk)-blocking experiments, MCs were blocked with piceatannol 10 μM for (Invivogen) for 15 min at 37°C. MCs were washed once before infection to remove excessive inhibitor.

Blood was obtained from the blood bank of the Norrland University Hospital Umeå anonymously following recommendations from the local ethical committee—Regionala etikprövningsnämnden i Umeå. Cord-blood donations were obtained from umbilical cord blood collected from normal, full-term deliveries at the Karolinska University Hospital. All donors were healthy donors and participants provided informed consent. Neutrophils were isolated from venous blood as previously described (Hosseinzadeh et al., 2012) and monocytes were isolated from buffy coats. Briefly, buffy coat blood was layered over equal amounts of Histopaque (ThermoScientific) and centrifuged at 900 rpm for 60 min. Leukocytes were collected and washed in PBS. Cells were then counted and diluted to 1 × 10^6^ cells/ml in RPMI and added to cell culture flask. After 24 h non-adherent cells were removed and the cell culture flask washed with fresh RPMI. Adherent cells were collected using a cell scraper.

### MC Granular Content Release

N-acetyl-beta-D-hexosaminidase and tryptase release was measured in HMC-1 cells (1 × 10^5^ cells/well) infected in a 96-well plate with *C. neoformans* or left untreated. After 1 h, 60 μL of supernatant was transferred to a 96-well plate and mixed with an equal volume of 7.5 mM of p-nitrophenyl-N-acetyl-β-D-glucosaminide dissolved in 80 mM of citric acid, pH 4.5. The mixture was incubated shaking for 2 h at 37°C. After incubation, 120 μL of glycine (0.2 M, pH 10.7) was added and absorbance was measured in a plate reader (Fluostar, BMG Labtech). Tryptase was measured using the mast cell degranulation kit (Millipore) according to the manufacturer's instructions.

For cytokine release, MCs (1 × 10^6^ cells/well) were infected with *C. neoformans* (MOI 5) for 20 h in 24-well plates at 37°C and 5% CO_2_. *C neoformans* only and uninfected MCs served as controls. At endpoint, the plate was centrifuged and supernatants collected and stored at −80°C. Supernatants were tested for the release of the following cytokines: IL-1β, IL-1ra, IL-2, IL-4, IL-5, IL-6, IL-7, IL-8, IL-9, IL-10, IL-12, IL-13, IL-15, IL-16, IL-17, IL-18, IL-5, Eotaxin, FGF Basic, G-CSF, GM-CSF, IFN-γ, IP-10, MCP-1, MIP-1a, PDGF-BB, MIP-1β, RANTES, TNF-α, VEGF, IL-1 α, IL-2RA, IL-12, CTACK, GROα, HGF, IFN-α2, LIF, MCP-3, M-CSF, MIF, MIG, β-NGF, SCF, SCGF-β, SDF-1 α, TNF-β, TRAIL using a Bio-Plex human cytokine assay (Bio-Rad Laboratories). Cytokine fold change was calculated as the ratio between infected condition and uninfected control. Additionally, MCP-1 release was confirmed using Human MCP-1 ELISA max deluxe (Biolegend) according to manufacturer's instructions.

### Fungal Killing

MCs (1 × 10^5^ cell/well) were infected with *C. neoformans* (MOI 5) in a 24-well plate. After 3 h, supernatants were plated in YEPD plates in triplicate. The fungal killing was measured as the number of fungal colonies forming units (CFU).

### Chemotaxis

Using a transwell system, as previously described (Lopes et al., 2015), the chemotactic migration of neutrophils (5 × 10^5^ cells/well) and monocytes (5 × 10^5^ cells/well) was tested with supernatants of MCs (1 × 10^5^ cell/well) infected with *C. neoformans* (MOI 5), uninfected controls or the equivalent number of fungal cells. Neutrophil migration was accessed for 30 min and monocyte migration for 90 min. Tested supernatants were collected and stored similarly as described for cytokine release.

### Phagocytosis

MCs phagocytosis of capsular and acapsular *C. neoformans* yeasts was performed according to Alanio et al. (2011) with slight modifications. Yeast cells (5 × 10^6^ /ml cells) were stained with IgG-Alexa-488 dye (A11006 Invitrogen) for 20 min and then centrifuged at 3,000 g for 10 min. Mast cells (1 × 10^6^ cells/ml) and *C. neoformans* were seeded to a flat-bottomed 24-well plate and incubated in a CO_2_ incubator at 37°C for 1 and 2 h. At the end-point, the mixture was collected to 1.5 ml eppendorf tubes and IgG-Alexa-633 dye (A21086 Invitrogen) was added for 20 min. The tubes were then centrifuged at 1,500 g for 10 min and the pellet re-suspended in cold deionized water for 10 min. The tubes were centrifuged at 1,500 g for 10 min and the pellet re-suspended in 1x PBS and measured using a FACS Accuri C6 instrument (BD Biosciences) with post-acquisition data analysis with Accuri C6 analysis. Phagocytized cryptococci were determined as the % of IgG-Alexa-488 dye high/IgG-Alexa-633 low cells in the FACS plot.

### Cell Death

MCs (5 × 10^4^ cells/well) were infected with *C. neoformans* (MOI 5) and host cell death was quantified using a sytox green-based (ThermoScientific) cell viability assay as described previously (Lopes et al., 2015). To measure the effect of cryptococcal mediators released into solution during delay of MC death we incubated *C. neoformans* for 20 h in RPMI medium. Cultures were centrifuged at 3,000 rpm for 10 min and the supernatants collected. Conditioned media containing 5, 10, 25, or 50% of overnight supernatants + fresh RPMI was incubated with MCs in the presence of sytox green.

### Statistical Analysis

All data are shown as mean ± SD. Significance in degranulation and cytokine release experiments was analyzed by multi-comparison using one-way ANOVA with Bonferroni post-test. Cell death experiments were analyzed applying two-way ANOVA with Bonferroni's post-test. Syk-inhibition and fungal killing experiment significances were analyzed using unpaired *t*-test with Welch correction. For all analyses, *p*-values < 0.05 were considered statistically significant.

## Results

### *C. neoformans* Triggers MC Degranulation

**Figure 1:**
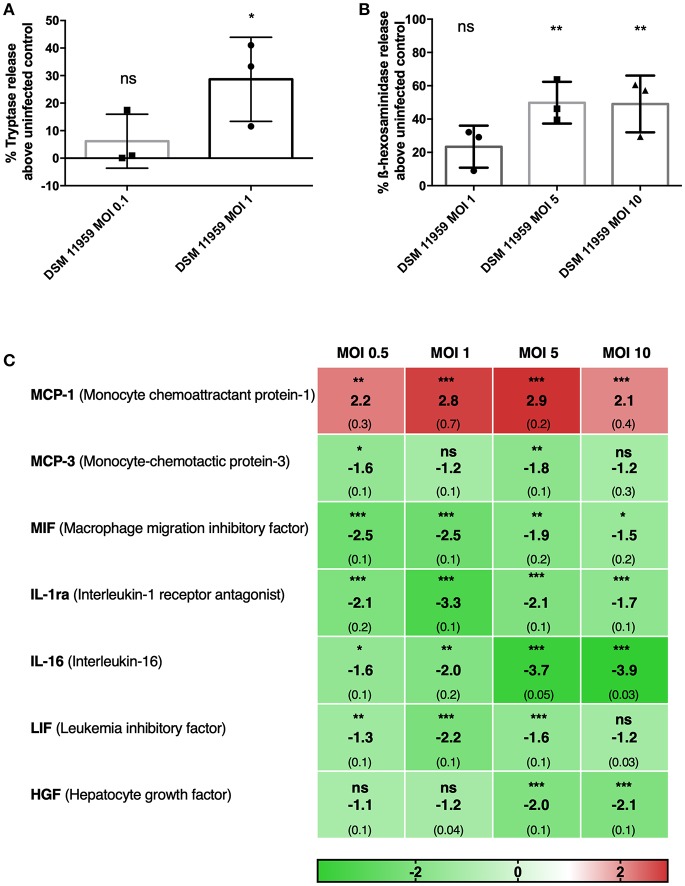
MCs show direct action against *C. neoformans* and release granular proteins. **(A)** MCs released tryptase after 6 h of infection with *C. neoformans* (MOI 1). Data from *n* = 3 (3). **(B)** MCs released ß-hexosaminidase after 1 h infection with *C. neoformans* (MOI 5 and 10). Data from *n* = 3 (3). **(C)** Fold change of a total of 48 cytokines were measured in supernatants of MCs infected with *C. neoformans* at different MOIs. Seven cytokines were differentially expressed and only MCP-1 was upregulated. Fold change was calculated as the ratio between infected condition and uninfected control. Data from *n* = 2 (4). For all analyses significance was analyzed by multi-comparison using one-way ANOVA with Bonferroni post-test. ^*^P ≤ 0.05; ^**^P ≤ 0.01; ^***^P ≤ 0.001; ns, not significant.

Secretion of granules containing inflammatory molecules is known to be one of the primary mechanisms employed by MCs. Degranulation elicits inflammatory reactions and recruits other immune cells (Beghdadi et al., 2011; Dudeck et al., 2018). MCs have recently been shown to degranulate in response to a component of the fungal cell, β-glucan, with a unique response pattern with included release of histamine and β-hexosaminidase but not LTC4, IL-6, or CCL2 (Barbosa-Lorenzi et al., 2017). We first assessed the degranulation of MCs in response to *C. neoformans* using tryptase and β-hexosaminidase quantification in supernatants of *in vitro* infection (Figures 1A,B). *C. neoformans* triggered MCs to release of tryptase and β-hexosaminidase in a dose-dependent manner. At MOI 1, MCs degranulated tryptase 30% above control levels (Figure 1A). Degranulation of β-hexosaminidase increased by 20% at MOI 1 and 50% at MOI 5 above control levels (Figure 1B).

To get a grip on effector molecules released from MCs released upon *C. neoformans* infection, we collected supernatants after 20 h of incubation and performed multiplex cytokine assays for 48 different molecules. Surprisingly, from the 48 tested cytokines, only MCP-1 was released upon *C. neoformans* stimulation (Figure 1C). MCP-1 is one of the key chemokines that regulate migration and infiltration of monocytes/macrophages to the tissues in response to infection (Deshmane et al., 2009). Since macrophages play an important role in anti-cryptococcal defense, we used MCP-1 secretion as a relevant readout for subsequent assays assessing MC activation upon encounter with *C. neoformans*. Cytokines released to a significantly lower extent than without stimulus encompassed MIF, MCP-3, IL-1ra, IL-16, LIF, and HGF.

**Figure 2:**
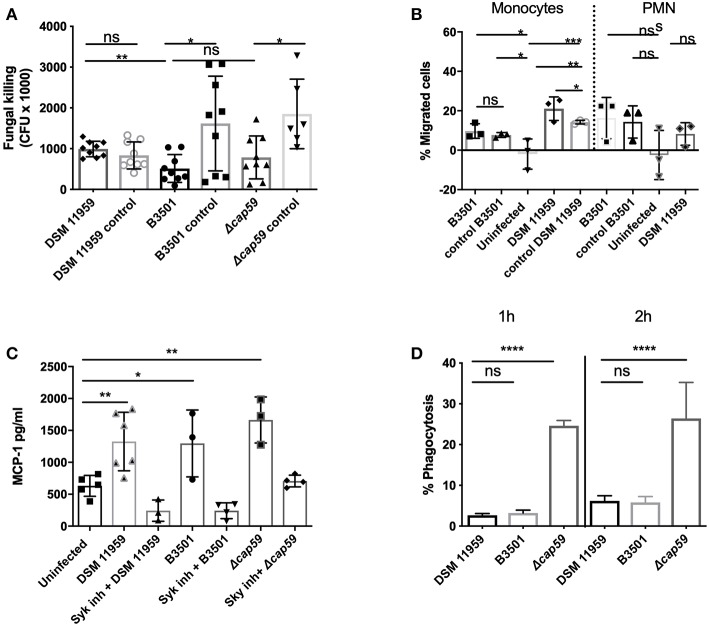
MC recognition of *C. neoformans* was mediated by Syk signaling and chemoattracts monocytes. **(A)** MCs induced killing was serotype dependent. Serotype A (DSM 11959) resisted killing by MCs whereas serotype D (B3501) and acapsular mutant cap59 showed some level of susceptibility. Data from *n* = 9. **(B)** Monocytes but not neutrophils were recruited by MCs after *C. neoformans* infection. The graph depicts the percentage of migration of neutrophil or monocyte migration over time toward supernatants of MCs infected with *C. neoformans*. **(C)** MCs secreted MCP-1 in a Syk dependent manner after 20 h infection with *C. neoformans* (MOI 5). MCP-1 was measured by ELISA in supernatants of HMC-1 infected with *C. neoformans* (MOI 5). Data from *n* = 3 (3). Syk was inhibited by piceatannol 10 μM after infection with HMC-1 infection with *C. neoformans* strains. **(D)** Acapsular mutants induce significantly more phagocytosis after 1 and 2 h infection. MC phagocytosis of *C. neoformans* was determined by FACS as the percentage of IgG-Alexa-488 dye high/IgG-Alexa-633 low *Cryptococcus* cells. Data from *n* = 4. Significance was analyzed by multi-comparison using one-way ANOVA with Bonferroni post-test in **(B,D)**. For **(A,C)** significance was analyzed using unpaired *t*-test with Welch correction. ^*^P ≤ 0.05; ^**^P ≤ 0.01; ^***^P ≤ 0.001; ^****^P ≤ 0.0001; ns, not significant.

To investigate if the release of MC mediators could contribute to the clearance of *C. neoformans*, we infected MCs with *C. neoformans* for 3 h and quantified the viability of yeast cells by plating (Figure 2A). We tested *wild-type* strain DSM 11959 and saw no reduction on the fungal load after incubation with MCs. To exclude if the presence of the capsule could disturb the killing, we used acapsular mutant cap59 and parental strain B3501. The viability of both strains was reduced approximately by 3-fold but surprisingly no statistical difference between the killing of the capsular strain and the acapsular mutant was seen. We propose that the difference between the two *wild-type* strains might arise from different susceptibility between different *C. neoformans* serotypes—DSM 11959 (serotype A) and B3501 (serotype D).

We conclude that MCs sense soluble components of *C. neoformans* and respond in a direct manner by releasing granular effectors and cytokines. The antifungal potential of these responses is dependent on the serotype of encountered *C. neoformans* strains.

### Monocytes but Not Neutrophils Are Chemoattracted in Response to Infection of MCs

Since MCs can modulate the recruitment of immune cells, we followed up on the ability of the chemokines secreted from infected MCs to induce migration of neutrophils and monocytes. In a functional migration assay we fluorescently labeled neutrophils or monocytes, added them to the upper compartment of a transwell system and allowed them to migrate toward the lower compartment containing supernatants from MCs infected with *C. neoformans*. In agreement with the exclusive induction of MCP-1 release, supernatants of infected MCs were chemotactic for monocytes but not for neutrophils, as only monocytes showed increased migration toward the lower compartment as compared to control samples (Figure 2B). Specific migration in response to the infection supernatants doubled as compared to migration toward supernatants from fungal cells alone. This indicates that MC degranulation triggered by *C. neoformans* selectively induced monocyte recruitment to the site of infection.

### MCs Recognize *C. neoformans* in a Syk-Dependent Manner

MCs use PRR to recognize fungal pathogen-associated molecular patterns (PAMPs). Most commonly, PRR ligands are fungal cell wall components, such as β-glucan. C-type lectin receptors (CLRs) are PRRs particularly important for the recognition of fungal PAMPs, for instance, CLR dectin-1 binds β-glucan (Shiokawa et al., 2017). Ligand binding to the C-type lectin receptors—dectin-1 and dectin-2 and mincle—triggers recruitment and phosphorylation of spleen tyrosine kinase (Syk) (Plato et al., 2013).

To test the role of CLRs in MC recognition of *C. neoformans* we blocked Syk activation in MCs by using specific inhibitor piceatannol using MCP-1 release as a readout (Oliver et al., 1994). Syk inhibition reduced MCP-1 release to basal levels suggesting that MC recognition of *C. neoformans* and release of MCP-1 is dependent on the signaling cascades involving CLRs and Syk (Figure 2C).

### *C. neoformans* Capsule Is Protective Against MC Phagocytosis

The polysaccharide capsule is an essential virulence factor and plays an important role in the interaction of this fungus with host immune recognition and response pathways (Alspaugh, 2015). In order to study the effect of the capsule of *C. neoformans* during MC phagocytosis, we incubated *C. neoformans* with MCs in a 24-well microtiter plate for 1 h and 2 h (Figure 2C). All *C. neoformans* cells were pre-labeled before infection and extracellular cells were additionally labeled at the end of the experiment. After successful MC lysis and FACS analysis, the percentage of intracellular yeast (single-labeled cells) could be determined.

Percentage of phagocytosis of cells from the capsular strain was 2% (DSM 11959) and 3% (B3501) at 1 h increasing to 5% (DSM 11959) and 7% (B3501) at 2 h. Cells from the acapsular mutant strain on the other hand where phagocytosed at much higher rates −25% at 1 h with an increase to 27% at 2 h. We concluded that the *C. neoformans* capsule protects against MC phagocytosis.

### Both Capsule and Soluble Factors of the *C. neoformans* Membrane Contribute to MC Survival

**Figure 3:**
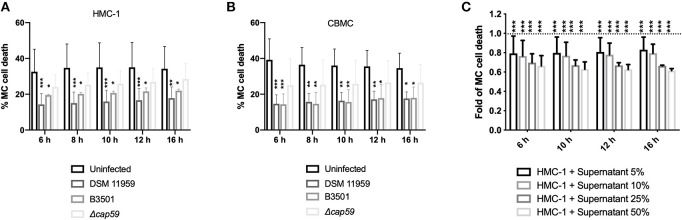
*C. neoformans* infection increase MC survival. **(A)** Infection with capsular *C. neoformans* increased HMC-1 survival. Infections were performed using MOI of 5. Data from *n* = 6 (3). **(B)** Infection with capsular *C. neoformans* (MOI 5) increased viability of primary MCs. Data from *n* = 4 (3). **(C)** Secreted components from *C. neoformans* overnight cultures induced MC survival. Plotted is the delayed death of resting MCs incubated with increasing concentrations of supernatants of an overnight culture of *C. neoformans* plotted as fold change in relation to untreated MCs (dashed line). For all analyses significance was analyzed by multi-comparison using two-way ANOVA with Bonferroni post-test. ^*^P ≤ 0.05; ^**^P ≤ 0.01; ^***^P ≤ 0.001; ^****^P ≤ 0.0001; ns, not significant.

To quantify MC death induced by *C. neoformans* we used a microplate-based fluorescence assay and the membrane-impermeable fluorescent DNA dye sytox. Fluorescent signal by DNA staining only occurs in cells with compromised plasma membranes. Cell death was calculated as the percentage of lysis control. Unexpectedly, both capsular *C. neoformans* strains promoted MC survival as uninfected MCs died half as much than infected cells (Figure 3A). Infection with acapsular mutants caused higher levels of death in MCs that were nevertheless not distinct from the death rate of uninfected controls (Figure 3A). We obtained similar results using primary MCs (Figure 3B). We concluded that *C. neoformans* capsule was important for modulation of the MC lifespan albeit not being the only factor responsible for that.

*C. neoformans* is known to secrete vesicles into a culture containing virulence factors and other cargo (Wolf et al., 2014). We reasoned that fungal secretion of extracellular factors could additionally help postpone MC death. To test our hypothesis, we incubated supernatants from an overnight culture of *C. neoformans* in different dilutions with fresh medium and incubated with resting MCs (Figure 3C). Increasing concentration of fungal conditioned supernatants partially delayed MC death. When using 50% fungal conditioned media MC cell death was decreased to 65% of uninfected baseline.

Overall, we concluded that MC lifespan is controlled by both by capsule presence and by the release of soluble fungal components.

## Discussion

*C. neoformans* is one of the most important causative agents of cryptococcosis and one of the major fungal pathogens. Yearly, *C. neoformans* is responsible for over 180 000 deaths worldwide affecting especially populations in Sub-Sahara (Rajasingham et al., 2017). Cryptococcosis is strongly associated with groups at risk to acquire severe opportunistic infections, such as HIV/AIDS patients (Rodrigues, 2016; Molloy et al., 2017).

Here, we explored the role of MCs in antifungal immunity against *C. neoformans*. MCs are important actors to maintain the equilibrium in host mucosa with significant contributions to symptoms caused by hypersensitivity and inflammatory reactions. Owing to the variety of expressed receptors MCs can carry out effector and regulatory tasks reacting to external stimuli (Halova et al., 2018). MCs are present in the oral mucosa of the nasal cavity and of the lungs and are therefore some of the most common cells to interact with *C. neoformans*. Using an *in vitro* model, we studied the interaction between MCs and *C. neoformans*. We applied different approaches to qualitatively and quantitatively assess this interplay.

Quantification of released mediators known to be produced by MCs in order to orchestrate immune reactions (Fukuishi et al., 2014; Metcalfe et al., 2016), such as β-hexosaminidase, tryptase, and cytokines, revealed that MCs recognized and responded to *C. neoformans* (Figures 1A,B).

Analysis of the cytokine multiplex showed that out of 48 cytokines only 7 were differentially secreted upon *C. neoformans* infection. From those only MCP-1 was released in a dose-dependent manner, whereas the release of six other cytokines and chemokines was reduced. *C. neoformans* infection has been shown to stimulate the production of MCP-1 in the lungs (Huffnagle et al., 1995) and failure to induce MCP-1 resulted in a diminished cell-mediated inflammatory response *in vivo* and diminished cryptococcal clearance (Huffnagle et al., 1995; Levitz et al., 1997). Three other cytokines are involved in leukocyte recruitment: MIF and MCP-3 are important for the recruitment of monocytic cells, whereas IL-16 is important for lymphocyte migration. The remaining three - IL-1ra, LIF, and HGF—are important in the regulation of inflammation. If the cytokine downregulation can be attributed to the direct action of the fungus remains to be determined but *C. neoformans* capsule polysaccharide has been previously implicated in cytokine downregulation in human monocytes (Vecchiarelli et al., 1995) while *C. gattii* downregulates the expression of antimicrobial peptide, cathelicidin, in human PBMCs (Herkert et al., 2018). Interestingly, in a previous study, we measured the response of MCs to *C. albicans* and detected a very different pattern of cytokine release (Lopes et al., 2018). Hence, MCs seem to orchestrate cytokine and chemokine responses dependently of the encountered fungal pathogen.

We tested the chemotactic potential of MC supernatants from infected cells. These supernatants induced migration of macrophages but not of neutrophils (Figure 2B). Neutrophils, besides being effective killers of fungi, constitute the second line of defense against *C. neoformans* behind alveolar macrophages (Mambula et al., 2000; Chiller et al., 2002) which are essential for fungal clearance during cryptococcosis (Hardison et al., 2012; Campuzano and Wormley, 2018). The pathogen has however evolved strategies to survive and replicate inside the macrophages and even use the host cell as a Trojan horse for dissemination (Geunes-Boyer et al., 2009; Casadevall, 2010; Davis et al., 2015). Our data suggest that *C. neoformans* also exploits MC responses in this regard since monocytic cells were selectively recruited.

Recognition of *C. neoformans* by MCs was dependent on Syk signaling (Figure 2C). This is in good agreement with a recent report describing non-opsonic uptake of cryptoccoci to be dependent on CLRs and its adaptor molecule Syk (Lim et al., 2018). Syk signaling is also know to be required for chemotaxis, helping in the reorganization of the actin cytoskeleton in response to chemokines (Park and Cox, 2011).

Antifungal activity of MCs was dependent on the fungal serotype since one *wild-type* strain was virtually resistant against MCs (Figure 2A). Alanio et al. showed that different clinical isolates of *C. neoformans* evoked varying macrophage responses contributing to different disease outcomes (Alanio et al., 2011). Notably, the presence of the capsule made no difference to the killing activity of MCs (Figure 2A). The killing of *C. neoformans* by neutrophils is also not influenced by the size or presence of the capsule but rather by the melanin-producing capacity of the fungus (Qureshi et al., 2011).

Moreover, *C. neoformans* infection delayed MC death as demonstrated by using both a cell line and primary cells (Figures 3A,B). Delaying host cell apoptosis is a mechanism used by other microbes to allow time for replication and cell-to-cell spread (Clifton et al., 1998). In this context, the immunomodulatory effect could reflect the ability of the fungus to augment immune responses mediated by MCs. Increased monocytic migration might allow the fungal cell to subsequently exploit incoming monocytic cells as a reservoir for dissemination. However, inhibition of apoptosis leading to *in vitro* survival of immune cells in response to β-glucan may also augment immune defense (Garcia-Valtanen et al., 2017).

The delay of MC death is larger when using capsular strains indicating a contribution of the polysaccharide capsule. Nevertheless, acapsular strains had similar death rates as uninfected MCs suggesting a complementary mechanism of modulation of MC lifespan (Figures 3A,B). We, therefore, decided to explore, if released fungal mediators could additionally contribute to delayed cellular death of MCs. Indeed, secretion of soluble mediators as culture supernatants helped to additionally mediate the effect (Figure 3C). The release of extracellular vesicles containing virulence factors is a described approach used by the fungus to modulate immune cell function (Rodrigues et al., 2007; Oliveira et al., 2010). During replication of *C. neoformans* in macrophages, accumulation of vesicles in the cytoplasm occurs (Tucker and Casadevall, 2002; Davis et al., 2015), which have immune modulatory effects (Ellerbroek et al., 2004; Bielska et al., 2018). Besides containing the major capsular polysaccharide, glucuronoxylomannan (GXM), other molecules such as sterylglucosidase are also present in these vesicles. Vesicles enriched in both components have been shown to delay the acute lethality *C. neoformans* infection (Colombo et al., 2019). Identification of the responsible soluble factors is currently under investigation.

Taken together, we show a new role for MCs during cryptococcosis. MCs influence the mounting of innate immune responses and can contribute to clearance. *C. neoformans*, in turn, might exploit the immune response aiming for dissemination. These findings highlight the complexity of MC and *C. neoformans* interaction. Further studies are needed to characterize the function of MCs during cryptococcosis in more detail, to find new targets for improved disease prevention strategies.

## Data Availability

The raw data supporting the conclusions of this manuscript will be made available by the authors, without undue reservation, to any qualified researcher.

## Ethics Statement

Blood was obtained from the blood bank of the Norrland University Hospital Umeå anonymously following recommendations from the local ethical committee—Regionala etikprövningsnämnden i Umeå. Cord-blood donation were obtained from umbilical cord blood collected from normal, full-term deliveries at the Karolinska University Hospital. All donors were healthy donors and participants provided informed consent.

## Author Contributions

CU conceived and designed the study. JL, MS, EB, and SH performed the experiments and analyzed the data. ME and GN provided materials and expertise. JL and CU wrote the manuscript.

### Conflict of Interest Statement

The authors declare that the research was conducted in the absence of any commercial or financial relationships that could be construed as a potential conflict of interest.
